# Molecular heterogeneity in malignant peripheral nerve sheath tumors associated with neurofibromatosis type 1

**DOI:** 10.1186/1479-7364-6-18

**Published:** 2012-09-04

**Authors:** Laura Thomas, Victor-Felix Mautner, David N Cooper, Meena Upadhyaya

**Affiliations:** 1 Institute of Medical Genetics, School of Medicine, Cardiff University, Cardiff, CF14 4XN, UK; 2 Department of Maxillofacial Surgery, University Medical Centre Hamburg-Eppendorf, Hamburg, D-20246, Germany

**Keywords:** Neurofibromatosis type 1, Malignant peripheral nerve sheath tumors, Molecular heterogeneity, *TP53*

## Abstract

Neurofibromatosis type-1 (NF1), resulting from *NF1* gene loss of function, is characterized by an increased risk of developing benign and malignant peripheral nerve sheath tumors (MPNSTs). Whereas the cellular heterogeneity of NF1-associated tumors has been well studied, the molecular heterogeneity of MPNSTs is still poorly understood. Mutational heterogeneity within these malignant tumors greatly complicates the study of the underlying mechanisms of tumorigenesis. We have explored this molecular heterogeneity by performing loss of heterozygosity (LOH) analysis of the *NF1*, *TP53*, *RB1*, *PTEN*, and *CDKN2A* genes on sections of 10 MPNSTs derived from 10 unrelated NF1 patients. LOH data for the *TP53* gene was found to correlate with the results of p53 immunohistochemical analysis in the same tumor sections. Further, approximately 70% of MPNSTs were found to display intra-tumoral molecular heterogeneity as evidenced by differences in the level of LOH between different sections of the same tumor samples. This study constitutes the first systematic analysis of molecular heterogeneity within MPNSTs derived from NF1 patients. Appreciation of the existence of molecular heterogeneity in NF1-associated tumors is important not only for optimizing somatic mutation detection, but also for understanding the mechanisms of NF1 tumorigenesis, a prerequisite for the development of specifically targeted cancer therapeutics.

## Introduction

Neurofibromatosis type 1 (NF1) (MIM 162200) is an autosomal dominant disorder affecting approximately 1 in 4,000 people worldwide [1]. NF1 is characterized by variable clinical features including benign cutaneous neurofibromas and plexiform neurofibromas in addition to pigmentary abnormalities comprising *café-au-lait* macules, Lisch nodules of the iris, and axillary and inguinal freckling. Learning difficulties and orthopedic problems also occur in up to 50% of individuals with NF1 [2]. Malignant complications are a less frequent but potentially much more serious manifestation of NF1. These often lead to premature death in individuals with NF1 and include malignant peripheral nerve sheath tumors (MPNSTs) which occur in approximately 10% to 15% of patients [3], pheochromocytomas, brain tumors, optic gliomas, gastrointestinal stromal tumors, and breast carcinomas [4].

The *NF1* gene, located at 17q11.2, encodes neurofibromin, a 2818 amino acid protein which is expressed at a high level in the brain and central nervous system [4]. Neurofibromin is a highly conserved RAS-GAP which negatively regulates Ras signaling [5-7] and the multiple downstream effectors activated by Ras, such as the PI3K and the MAPK (mitogen-activated kinase) signaling cascades that are involved in regulating cell proliferation, DNA synthesis, and apoptosis. The loss of neurofibromin function due to *NF1* gene inactivation therefore results in the constitutive activation of many of these down-regulated systems leading to increased cell proliferation and an increased likelihood of tumorigenesis.

In line with the tumor suppressor role of the *NF1* gene, mutational inactivation of both NF1 alleles is required to change the phenotype of the cell: a first (inherited) mutation in one *NF1* allele is followed by the somatic loss of the remaining wild-type *NF1* allele via a number of different mutational mechanisms. Loss of heterozygosity (LOH), for example, represents a common form of loss of function of the wild-type *NF1* allele in somatic cells such as Schwann cells which form neurofibromas, owing to the presence of an inherited *NF1* gene lesion on the other allele. [8,9] LOH is known to be caused by a number of mechanisms including deletions of genetic material and the loss of a whole chromosome by nondisjunction with or without reduplication. However, mitotic recombination has been demonstrated to be the most common event accounting for LOH in NF1-associated tumors [9].

In addition to *NF1* gene mutations, a number of other loci are also known to be involved in the process of NF1 tumorigenesis. Thus, additional somatic mutations have been identified in NF1-associated MPNSTs that affect several genes encoding proteins involved in cell cycle regulation: *TP53, RB1CDKN2A*[10-17], and *PTEN*[18]. Alterations in *TP53* and *RB1* have also been identified in plexiform neurofibromas [19] and more recently in cutaneous neurofibromas from patients with a high tumor burden [20]. These genetic alterations act so as to promote cellular proliferation as a consequence of *CDKN2A* loss and to bring about abnormal cell cycle arrest mediated by DNA damage and aberrant apoptosis as a consequence of the loss of *TP53* and *RB1.*

Tumor heterogeneity is a notoriously challenging aspect of cancer biology, responsible for introducing very significant complexity into both the study of the underlying mechanisms of tumor development and the therapeutic context [21-23]. Tumor heterogeneity has been modeled using a number of different biomarkers to identify the extent of heterogeneity at the cellular, molecular, and genome architectural levels [24,25]. Cellular heterogeneity is a well-established feature of NF1-associated tumors, particularly in benign cutaneous neurofibromas in which only the Schwann cells harbor *NF1* mutations, although the tumors also contain fibroblasts, mast cells, perineural cells, and axons [26]. The tumor microenvironment is also known to impact upon the development of NF1-associated tumors, with mast cells a likely contributor to neurofibroma development [27]. As with benign neurofibromas, malignant NF1-associated tumors are known to be heterogeneous in nature, invariably containing diverse subpopulations of tumor cells, including benign and malignant cells, fibroblasts, and infiltrating inflammatory cells [26]. At the molecular level, malignant tumors are recognized as being highly heterogeneous in terms of both their accumulated genetic mutations and their phenotypic *CDKN2A, TP53, RB1* expression profiles [13,15,16].

Analysis of molecular and cellular heterogeneity using a variety of methods promises to generate important new insights into tumor biology as well as the underlying processes of tumorigenesis. This is the first study to comprehensively determine the level of molecular heterogeneity in a panel of MPNSTs derived from patients with NF1. It is hoped that this will provide further understanding of the molecular heterogeneity of malignant tumors in the context of the underlying background cellular and genome architectural heterogeneity. Furthermore, such studies may allow both therapeutic sensitivities and the efficacy of potential drug treatments to be comprehensively evaluated.

## Materials and methods

### Patients

Ten unrelated patients, all exhibiting the NIH diagnostic criteria for NF1 (reviewed by Upadhyaya [1]) were analyzed in this study. Germline mutations have been detected in 7 of the 10 patients (Table 1); however, full clinical details were not available from all patients. A total of 10 MPNSTs derived from these patients were carefully and individually macrodissected to yield clean sections which were in turn further subdivided. The number and size of the various sections and subsections were mainly dependent upon the size of the original tumor (Table 1 and Additional file 1: Supplementary Figure S1). DNA was extracted from all sections of tumor samples and corresponding constitutional (blood) samples by means of the phenol/chloroform extraction method [28]. This study obtained approval from the appropriate institutional review boards. All patients involved provided their informed consent.

**Table 1:** **Analysis of molecular heterogeneity with respect to *NF1*,* TP53*,* RB1*,* CDKN2A*, and* PTEN* in 10 MPNSTs**

**Tumor**	**Tumor number**	**Tumor section**	**Tumor subsection**	***NF1***	***TP53***	***RB1***	***PTEN***	***CDKN2A***
1	T196.22	A	1	LOH	LOH	LOH	LOH	LOH
			2	LOH	LOH	LOH	LOH	LOH
			3	LOH	LOH	50% LOH	LOH	LOH
			4	1 2	1 2	1 2	1 2	1 2
			5	LOH	LOH	50% LOH	LOH	LOH
			6	LOH	LOH	LOH	LOH	LOH
			7	LOH	LOH	LOH	LOH	LOH
			8	LOH	LOH	50% LOH	50% LOH	LOH
			9	1 2	LOH	1 2	LOH	LOH
			10	1 2	LOH	1 2	LOH	LOH
		B	1	LOH	50% LOH	50% LOH	50% LOH	1 2
			2	LOH	50% LOH	50% LOH	50% LOH	1 2
			3	LOH	50% LOH	50% LOH	50% LOH	1 2
			4	LOH	50% LOH	50% LOH	50% LOH	1 2
			5	50% LOH	50% LOH	50% LOH	50% LOH	1 2
			6	LOH	50% LOH	50% LOH	50% LOH	1 2
			7	LOH	50% LOH	50% LOH	50% LOH	1 2
			8	LOH	50% LOH	50% LOH	50% LOH	1 2
			9	LOH	50% LOH	50% LOH	50% LOH	1 2
			10	LOH	50% LOH	50% LOH	50% LOH	1 2
		C	1	LOH	LOH	50% LOH	LOH	1 2
			2	LOH	LOH	50% LOH	LOH	1 2
			3	LOH	LOH	50% LOH	LOH	1 2
			4	LOH	LOH	50% LOH	LOH	1 2
			5	1 2	LOH	1 2	50% LOH	50% LOH
			6	LOH	LOH	50% LOH	LOH	50% LOH
			7	LOH	LOH	50% LOH	LOH	50% LOH
			8	LOH	LOH	50% LOH	LOH	50% LOH
			9	LOH	LOH	50% LOH	LOH	50% LOH
			10	LOH	LOH	50% LOH	LOH	50% LOH
		D	1	LOH	LOH	50% LOH	LOH	50% LOH
			2	LOH	LOH	50% LOH	50% LOH	1 2
			3	LOH	LOH	50% LOH	LOH	LOH
			4	LOH	LOH	50% LOH	LOH	LOH
			5	LOH	LOH	50% LOH	LOH	LOH
			6	LOH	LOH	50% LOH	LOH	LOH
			7	LOH	LOH	50% LOH	LOH	LOH
			8	LOH	LOH	50% LOH	LOH	LOH
			9	LOH	LOH	50% LOH	LOH	LOH
			10	LOH	LOH	50% LOH	LOH	LOH
		E	1	LOH	1 2	1 2	1 2	1 2
			2	LOH	1 2	1 2	50% LOH	1 2
			3	50% LOH	1 2	1 2	1 2	1 2
			4	LOH	1 2	1 2	50% LOH	1 2
			5	1 2	1 2	1 2	0%	1 2
			6	50% LOH	1 2	1 2	50% LOH	1 2
			7	50% LOH	1 2	1 2	0%	1 2
			8	LOH	1 2	1 2	1 2	1 2
			9	LOH	1 2	1 2	1 2	1 2
			10	LOH	1 2	1 2	1 2	1 2
2	T516	A	1	LOH	50% LOH	1 2	1 2	1 2
			2	1 2	50% LOH	1 2	1 2	1 2
		B	1	50% LOH	50% LOH	1 2	1 2	1 2
			2	50% LOH	50% LOH	1 2	1 2	1 2
		C	1	1 2	1 2	1 2	1 2	1 2
			2	1 2	1 2	1 2	1 2	1 2
3	T517	A	1	LOH	50% LOH	1 2	1 2	1 2
			2	LOH	50% LOH	1 2	1 2	1 2
		B	1	LOH	50% LOH	1 2	1 2	1 2
			2	1 2	1 2	50% LOH	1 2	1 2
4	T518	A	1	1 2	50% LOH	50% LOH	1 2	1 2
			2	LOH	1 2	50% LOH	1 2	50% LOH
		B	1	LOH	50% LOH	1 2	1 2	1 2
			2	1 2	1 2	1 2	1 2	1 2
		C	1	1 2	1 2	1 2	1 2	1 2
			2	1 2	1 2	1 2	1 2	1 2
5	T519	A	1	1 2	50% LOH	50% LOH	1 2	1 2
			2	50% LOH	50% LOH	50% LOH	1 2	1 2
		B	1	50% LOH	50% LOH	1 2	1 2	1 2
			2	50% LOH	50% LOH	50% LOH	1 2	1 2
6	T521	A	1	LOH	1 2	1 2	1 2	1 2
			2	LOH	1 2	1 2	1 2	1 2
		B	1	LOH	1 2	1 2	1 2	1 2
			2	LOH	1 2	1 2	1 2	1 2
7	T522	A	1	LOH	1 2	1 2	1 2	1 2
			2	LOH	1 2	1 2	1 2	1 2
8	T523	A	1	LOH	1 2	1 2	1 2	1 2
			2	LOH	1 2	1 2	LOH	1 2
9	T524	A	1	LOH	1 2	1 2	1 2	LOH
			2	LOH	1 2	1 2	1 2	LOH
10	T525	A	1	LOH	1 2	1 2	1 2	1 2
			2	LOH	1 2	1 2	1 2	1 2
		B	1	LOH	1 2	1 2	1 2	LOH
			2	LOH	1 2	1 2	1 2	LOH

LOH denotes samples in which approximately 100% LOH of the second allele has occurred; 50% LOH denotes samples in which approximately 50% of the second allele was lost; 1 2 denotes samples in which the marker was clearly present in the heterozygous state, denoting the absence of LOH. The results represent the summed data for all markers analyzed at each genetic loci (*NF1*, *TP53*, *RB1*, *CDKN2A* and *PTEN).*

### LOH analysis

LOH analysis was performed on all sections derived from the 10 MPNSTs and their corresponding lymphocyte DNA samples with a panel of fluorescently tagged microsatellite markers located within the five genes investigated: *NF1* (*D17S799J1J2IVS27EV120IVS383′NF1-1D17S798D17S250*, and *D17S1822*) [28]*, TP53* (D17S796, *TP53 InvTP53 c.72TP53 Exon 6D17S938*, and *D17S804*) [29,30]*, RB1* (*D13S118D13S153D13S917RB1.2RB1.26*, and *D13S119*) [31,32], *CDKN2A* (*D9S1751D9S942D9S304*, and *D9S748*) [33,34], and *PTEN* (*D10S2491D10S215*, exon 3, and exon 8) [35]. Molecular heterogeneity was assessed by noting differences in the level of LOH between different sections of the same tumor sample.

Markers were analyzed by means of an ABI 3100 genetic analyzer using Genotyper and Genescan software (Applied Biosystems, Warrington, UK) [28]. Allele loss in a tumor was scored if the area under one allelic peak in the tumor section was reduced relative to the other allele, after correction of the relative peak areas against the corresponding lymphocyte DNA sample. For LOH to be confirmed, at least two adjacent markers were required to exhibit a reduced signal by at least 50%. Samples were deemed to display LOH when complete (approximately 100%) loss of the second allele was evident. Samples exhibiting between approximately 50% and 99% loss of the second allele were termed ‘50% LOH’, whereas a marker which demonstrated no LOH (approximately 0% to 49%) was simply classed as heterozygous (‘1 2’).

### p53 Immunohistochemical analysis

Immunohistochemical (IHC) analysis of p53 was performed on five sections derived from five MPNSTs (T196.22, T516, T517, T518, and T519) which were found by LOH analysis to exhibit molecular heterogeneity with respect to the *TP53* gene (Table 1). Immunohistochemistry was performed using a standard protocol [9]. Briefly, 5-μm-thick slides were cut from blocks of formalin-fixed paraffin-embedded MPNST tissue using a microtome (Leica Microsystems, Wetzlar, Germany). Deparaffination was completed in ethanol and xylene prior to antigen recovery which was performed using boiling citrate buffer. Endogenous peroxidase was blocked with 3% hydrogen peroxide, while goat serum (Vector Labs, Peterborough, UK) was used in the blocking step. A 100-μl pre-diluted mouse anti-p53 monoclonal primary antibody (Launch Diagnostics, Kent, UK) was used as supplied and added to each slide and incubated at 4 °C overnight. Each slide was then incubated with 100 μl secondary antibody (Vector Labs) for 30 min and subsequently with 100 μl ABC peroxidase (Vector Labs) for 30 min. DAB (Vector Labs) was added to each slide to allow the color to develop. Slides were dehydrated and mounted with DPX (VWR).

## Results

### LOH analysis

**Figure 1:**
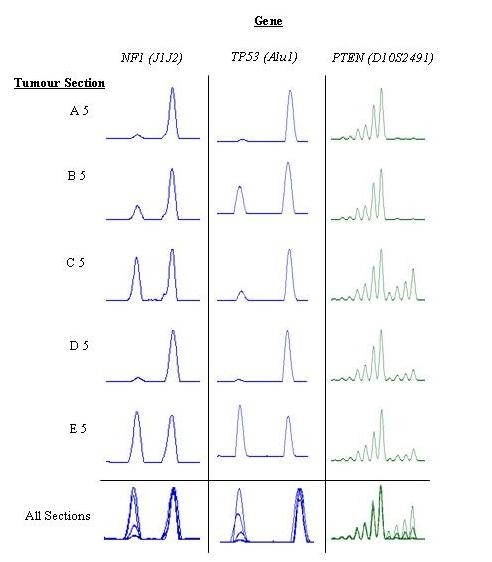
**LOH patterns in *NF1*, *TP53***, **and* PTEN* within sections A to E derived from T196.22.***All sections* represents the overlaid LOH results from all five sections (**A** to **E**) illustrating the different levels of gene-specific LOH observed within a single tumor. The results presented are representative of LOH at each genetic loci and are derived from individual markers analyzed at each of these loci (*NF1 - J1J2*, *TP53 - Alu1*, and *PTEN - D10S2491)*

Varying sizes of 10 MPNST tumors, derived from 10 different individuals, were independently sectioned, and the LOH of five genes (*NF1*, *TP53*, *RB1*, *CDKN2A*, and *PTEN*) was analyzed for each section. Germline mutations were detected in 7 of the 10 patients. Although this is a relatively small sample, the germline mutation does not appear to correlate with the level of molecular heterogeneity. LOH of the *NF1* gene was identified in all 10 tumors. In addition, LOH was noted for the *TP53* gene (five tumors: T196.22, T516, T517, T518, and T519), the *RB1* gene (four tumors: T196.22, T517, T518, and T519), the *CDKN2A* gene (four tumors: T196.22, T518, T524, and T525), and the *PTEN* gene (two tumors: T196.22 and T523) (Table 1, Figure 1, and Additional file 2: Supplementary Table S1). In only 2 of the 10 tumors (T521 and T522) was LOH not found in at least one of the four loci other than *NF1* (i.e. *TP53*, *RB1*, *CDKN2A* or *PTEN*). Further, 7 of the 10 tumors (70%) were found to exhibit intra-tumoral molecular heterogeneity with respect to at least one gene, as defined by a varying level of LOH within the same tumor (T196.22, T516, T517, T518, T519, T523, T525; i.e., between the subsections). Additional cytogenetic analysis was not available to confirm the LOH identified.

Some of the larger tumors (e.g., T521) were found to exhibit no molecular heterogeneity, whereas some smaller tumors (e.g., T523) displayed molecular heterogeneity albeit only with respect to a specific gene. Additionally, five of the tumors analyzed in this study were known to be high grade (T196.22, T517, T519, T522, and T525). Four of these high grade tumors in our cohort exhibited molecular heterogeneity (*viz*. T196.22, T517, T519, and T525), whereas such heterogeneity was absent in the remaining sample graded as high (T522). For the remaining samples, details on the grade of tumor were unknown. The extent of LOH in the *NF1* gene was also found to vary between sections of the same tumor. Thus, some sections displayed LOH over the entire *NF1* gene and surrounding regions, whereas other sections of the same tumor only exhibited LOH within a portion of the *NF1* gene. Details of the extent of LOH in each tumor are given in Additional file 2: Supplementary Table S1.

### p53 Immunohistochemical analysis

**Figure 2:**
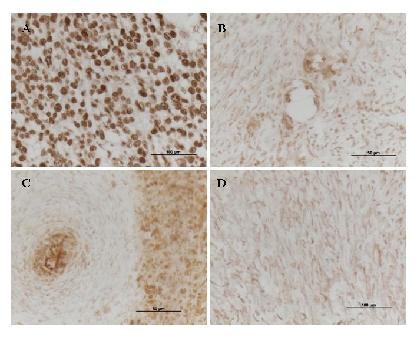
**Immunohistochemical analysis of p53 on section 5 B,C and E (T196.22).** (**A**) Positive control; breast carcinoma localized positive stain for p53. (**B**) T196.22,B5; slight localized positive p53 staining in a few cells. (**C**) T196.22,C5; localized positive and negative staining for p53. (**D**) T196.22,E5; Lack of p53 immunohistochemical staining

IHC analysis of the tissue distribution of p53 was found to correlate broadly with the results of the LOH analysis (Figure 2). Thus, different sections of the five MPNST tumors analyzed were found to display differences in staining for p53 that closely matched the presence or absence of LOH for the *TP53* gene (Table 1). For example, sections A, C, and D of T196.22 displayed complete LOH of the *TP53* gene in the majority of MPNST sections, and this was reflected in higher p53 expression as seen by IHC in these samples (Figure 2B,C). Conversely, section E (T196.22) showed no p53 expression by IHC peroxidase staining, and this section was found not to display any LOH (Figure 2D). Additionally, section B (T196.22) showed lower levels of staining (Figure 2B) than that of T196.22 section C (Figure 2C) but more than that of T196.22 section E (Figure 2D). These results correlate with those of the LOH analysis in which section T196.22 section B was found to have 50% LOH, but section T196.22 section C had 100% LOH and section E (T196.22) had no LOH. p53 Positivity by IHC has previously been reported in 60% of MPNSTs, whereas neurofibromas are p53 immunonegative [36].

## Discussion

Cellular heterogeneity is a well-known complication in the analyses of NF1-associated tumors. By contrast, heterogeneity at the molecular level in NF1-associated tumors has scarcely been addressed. In this study, we set out to determine the extent of molecular heterogeneity within and between 10 MPNSTs, derived from 10 unrelated NF1 patients, by determining the differences in the levels of LOH at the *NF1*, *TP53*, *RB1*, *CDKN2A*, and *PTEN* gene loci. The results of this study indicated that 70% of the MPNST tumors studied exhibit molecular heterogeneity between sections of the same tumor sample. This heterogeneity was especially evident in the case of those sections from the same tumor which were anatomically adjacent to each other prior to dissection but which had nevertheless been found to differ with respect to the degree of LOH. Indeed, some sections were found to be entirely devoid of LOH for all five gene loci analyzed but were located beside sections exhibiting complete LOH for one or more of these genes (e.g., T196.22 section A4). Prior to dissection, in a number of the MPNST tumor sections under study, there were clearly defined centrally located tumor portions (T196.22 subsection 5 A to E, T516 section B, T518 section B). These areas corresponded to the sections in which different levels of LOH and subsequently *TP53* staining were identified (T196.22 section B5, T196.22 section C5, T196.22 section E5; Figure 2).

The observation that *TP53* LOH correlates directly with the p53 expression, as demonstrated here, has been observed in previous studies [37,38]. However, the precise relationship between *TP53* LOH and p53 protein expression remains unclear since some studies have identified p53 expression in tumors which have no *TP53* LOH. This may be explicable in terms of the presence of two *TP53* alleles, one mutant and the other wild-type in a given cell type, resulting in the production of both wild-type and mutant p53 protein. As a consequence, p53 may be detected by IHC even in the absence of p53 function. This suggests that in the context of evaluating tumor heterogeneity, IHC analysis is unlikely to be as reliable as the other molecular genotyping methods. IHC analysis could therefore be replaced by more accurate methods including AQUA [39,40] and tissue analysis with multiplex quantum dots (QD) [25] to yield a digital map of molecular and cellular heterogeneity to improve the sensitivity of detection and the prediction of a therapeutic response.

p53 is associated with malignant transformation in NF1-associated tumors [18] and LOH of the *TP53* gene was identified in 5 of the 10 tumors under study (Table 1, Additional file 2: Supplementary Table S1). *TP53* has also been found to manifest in intra-tumoral molecular heterogeneity with respect to its mutation in other tumor types, including breast cancer [41,42]. In pancreatic cancer, molecular heterogeneity is evident in cells with different capacities for initiating metastasis [43] suggesting that molecular heterogeneity may well prove to be the rule rather than the exception. If the molecular heterogeneity identified in this set of tumors was to emerge as relevant to MPNST development, we may have to revise our view not only of MPNST tumor biology, but also of the basic processes underlying MPNST tumorigenesis.

It might be assumed that, owing to the large size of some of the MPNST tumors, they would be divisible into a larger number of sections thereby allowing molecular heterogeneity to be assessed more clearly. However, the size of the tumor was not found to correlate with the level of molecular heterogeneity detected. It is, however, possible that intra-tumoral molecular heterogeneity could be related in some way to the grade of tumor, at least for those tumor samples analyzed here. A larger study is clearly warranted in order to determine whether these results could be replicated in a larger set of NF1-associated MPNSTs.

The pathological diagnosis of an MPNST is usually held to represent the ‘gold standard’ for the purposes of analysis and currently relies on the examination of not just one but a number of different sections. The results of this study, and from other previous studies on solid tumors [44-46], are broadly illustrative of the importance of careful dissection in the analysis of large tumors and suggest that in the interest of diagnostic accuracy, molecular analysis should be performed on several tumor sections alongside a pathological diagnosis. The clear implication for those studies that involve microarray analysis is that replicates across several sections would be advisable.

The results of this study therefore have important implications for molecular studies of NF1-associated tumor specimens. For example, although molecular techniques currently employed in mutation detection in large solid tumors are adequate for identifying and characterizing the underlying molecular and genetic aberrations, the potential for molecular heterogeneity means that a single dissected piece of tumor should not be assumed to be representative of the tumor as a whole; as a consequence, some somatic mutations may well be missed.

Although MPNSTs only develop in approximately 15% of NF1 patients, they represent a frequent cause of lethal progression of the NF1 phenotype. The prognosis for individuals diagnosed with an MPNST is usually very poor; 5-year survival rates in patients with advanced non-resectable and/or metastatic MPNSTs that have exhibited a limited response to chemotherapy are in the order of 20% to 50%, while 10-year survival rates are as low as 7.5% [47]. Treatment options for MPNSTs are currently rather limited, and complete surgical excision with clear margins is the recommended therapy for MPNSTs. A larger study is required in order to determine the full extent of molecular heterogeneity within MPNSTs in NF1 patients. However, such a study will be laborious and time-consuming to set up especially as MPNSTs are quite rare.

Genomic instability and high intra-tumoral genetic heterogeneity may synergize so as to accelerate the evolutionary processes within the tumor leading to the development of resistance to cytotoxic and targeted anticancer drugs. Improvements clearly need to be made to the treatment regimes for patients with MPNSTs. The results from this study indicate that while drugs can be developed *in vitro* and in *vivo* animal studies that would be capable of targeting the genes involved in the genesis of MPNSTs, the efficacy of these drugs is likely to be somewhat limited unless the cellular, molecular, and architectural heterogeneity of the tumor are considered alongside the tumor microenvironment.

This study represents the first systematic analysis of molecular heterogeneity in MPNSTs associated with NF1. The molecular heterogeneity evident at a number of different gene loci indicates that there is an urgent need not only for the integration of molecular and morphological biomarkers in cancer diagnosis, but also for the development of specific treatments for NF1-associated MPNSTs.

## Competing interests

The authors declare that they have no competing interests.

## Authors’ contributions

LT performed the laboratory work and contributed to writing; V-FM provided the tumors; DNC contributed to writing; and MU conceived this study and provided overall directions. All authors read and approved the final manuscript.

## Supplementary Material

Additional file 1 **Supplementary figure 1. Example of macrodissection of tumor 1 (T196.22).** The tumor was divided into five large sections (A to E). These sections were then subdivided into 10 further sections (1 to 10). (PDF 21 kb) Click here for file

Additional file 2 **Supplementary table 1. Extent of LOH analysis in 5 genes (*NF1*, *TP53*, *RB1*, *CDKN2A* and* PTEN*) in 10 MPNSTs.** (PDF 522 kb) Click here for file
