# Supplementary figures and images for: Molecular heterogeneity in malignant peripheral nerve sheath tumors associated with neurofibromatosis type 1
Source: Hum Genomics. 2012 Sep 4;6(1):18. doi: 10.1186/1479-7364-6-18 (PMC3500234; doi:10.1186/1479-7364-6-18)

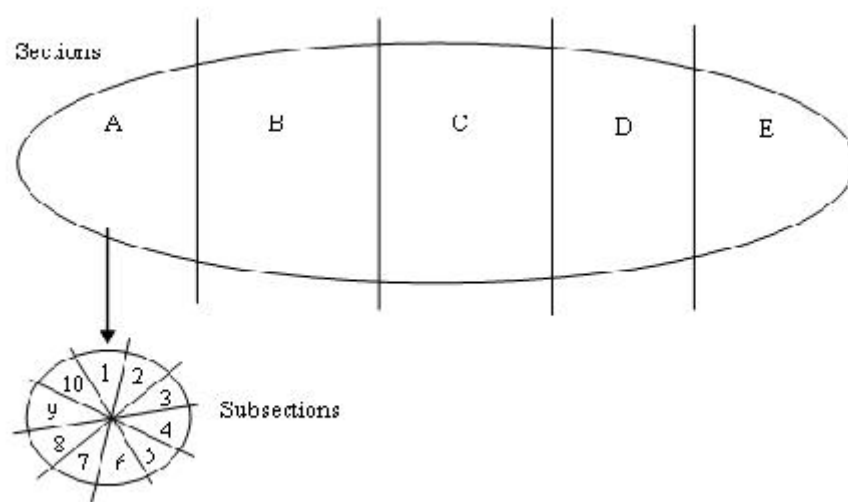

Supplementary Figure 1

Supplement: Additional file 1 — Supplementary figure 1. Example of macrodissection of tumor 1 (T196.22). The tumor was divided into five large sections (A to E). These sections were then subdivided into 10 further sections (1 to 10). (PDF 21 kb) [file 1479-7364-6-18-S1.pdf]
