# Supplementary material for: Molecular heterogeneity in malignant peripheral nerve sheath tumors associated with neurofibromatosis type 1
Source: Hum Genomics. 2012 Sep 4;6(1):18. doi: 10.1186/1479-7364-6-18 (PMC3500234; doi:10.1186/1479-7364-6-18)
Supplement: Additional file 2 — Supplementary table 1. Extent of LOH analysis in 5 genes (NF1, TP53, RB1, CDKN2A and PTEN) in 10 MPNSTs. (PDF 522 kb) [file 1479-7364-6-18-S2.pdf]

Supplementary Table 1

Tumour	Tumour Number	Gene Analysed and Extent of LOH				
		NF1	*TP53*	*RB1*	*PTEN*	*CDKN2A*
1	T196.22	LOH: D17S799-D17S1822 (17p11.2-17q25.2)	LOH: TP53 Inv-D17S938 (TP53 Intron 1-5'TP53)	LOH: D13S118-D13S119 (5'-3'*RB1*)	LOH: D10S215-D10S2491 (10q22-23-10q23-23)	LOH: D9S304-D9S748 (9p21.1-9q32)
2	T516	LOH: IVS27-IVS38 (Intron 27-38)	LOH: TP53 Inv-TP53 E:6 (TP53 Intron 1-Exon6)	No LOH	No LOH	No LOH
3	T517	LOH: IVS27-IVS38 (Intron 27-38)	LOH: TP53 Inv-TP53 E:6 (TP53 Intron 1-Exon6)	LOH: RB1.2-RB1.26 (Exon2-26)	No LOH	No LOH
4	T518	LOH: J1J2-3'NF1 (Intron 27-38)	LOH: TP53 Inv-TP53 E:6 (TP53 Intron 1-Exon6)	LOH: RB1.2-RB1.26 (Exon2-26)	No LOH	LOH: D9S304-D9S748 (9p21.1-9q32)
5	T519	LOH: IVS38-3'NF1 (Intron 38-3'*NF1*)	LOH: TP53 Inv-TP53 E:6 (TP53 Intron 1-Exon6)	LOH: D13S917-RB1.26 (5'RB1-Exon 26)	No LOH	No LOH
6	T521	LOH: J1J2-EV120 (Intron 27-38)	No LOH	No LOH	No LOH	No LOH
7	T522	LOH: D17S799-3’NF1 (17p11.2- 3'*NF1*)	No LOH	No LOH	No LOH	No LOH
8	T523	LOH: J1J2-D17S250 (Intron 27-3'*NF1*)	No LOH	No LOH	LOH: D10S215-D10S2491 (10q22-23-10q23-23)	No LOH
9	T524	LOH: J1J2-3'NF1 (Intron 27-38)	No LOH	No LOH	No LOH	LOH: D9S304-D9S748 (9p21.1-9q32)
10	T525	LOH: D17S799-D17S1822 (17p11.2-17q25.2)	No LOH	No LOH	No LOH	LOH: D9S304-D9S748 (9p21.1-9q32)
Total with LOH		10	5	4	2	4
